# The Hepatitis B Virus Ribonuclease H Is Sensitive to Inhibitors of the Human Immunodeficiency Virus Ribonuclease H and Integrase Enzymes

**DOI:** 10.1371/journal.ppat.1003125

**Published:** 2013-01-22

**Authors:** John E. Tavis, Xiaohong Cheng, Yuan Hu, Michael Totten, Feng Cao, Eleftherios Michailidis, Rajeev Aurora, Marvin J. Meyers, E. Jon Jacobsen, Michael A. Parniak, Stefan G. Sarafianos

**Affiliations:** 1 Department of Molecular Microbiology and Immunology, Saint Louis University School of Medicine, Saint Louis, Missouri, United States of America; 2 Saint Louis University Liver Center, Saint Louis University School of Medicine, Saint Louis, Missouri, United States of America; 3 Key Laboratory of Molecular Infectious Diseases, Ministry of Education, Chongqing Medical University, Chongqing, People's Republic of China; 4 Department of Infectious Diseases and Microbiology, University of Pittsburgh School of Public Health, Pittsburgh, Pennsylvania, United States of America; 5 Center for World Health and Medicine, Saint Louis University School of Medicine, Saint Louis, Missouri, United States of America; 6 Department of Molecular Microbiology and Immunology and Department of Biochemistry, University of Missouri School of Medicine, Christopher S. Bond Life Sciences Center, Columbia, Missouri, United States of America; University of California, San Diego, United States of America

## Abstract

Nucleos(t)ide analog therapy blocks DNA synthesis by the hepatitis B virus (HBV) reverse transcriptase and can control the infection, but treatment is life-long and has high costs and unpredictable long-term side effects. The profound suppression of HBV by the nucleos(t)ide analogs and their ability to cure some patients indicates that they can push HBV to the brink of extinction. Consequently, more patients could be cured by suppressing HBV replication further using a new drug in combination with the nucleos(t)ide analogs. The HBV ribonuclease H (RNAseH) is a logical drug target because it is the second of only two viral enzymes that are essential for viral replication, but it has not been exploited, primarily because it is very difficult to produce active enzyme. To address this difficulty, we expressed HBV genotype D and H RNAseHs in *E. coli* and enriched the enzymes by nickel-affinity chromatography. HBV RNAseH activity in the enriched lysates was characterized in preparation for drug screening. Twenty-one candidate HBV RNAseH inhibitors were identified using chemical structure-activity analyses based on inhibitors of the HIV RNAseH and integrase. Twelve anti-RNAseH and anti-integrase compounds inhibited the HBV RNAseH at 10 µM, the best compounds had low micromolar IC_50_ values against the RNAseH, and one compound inhibited HBV replication in tissue culture at 10 µM. Recombinant HBV genotype D RNAseH was more sensitive to inhibition than genotype H. This study demonstrates that recombinant HBV RNAseH suitable for low-throughput antiviral drug screening has been produced. The high percentage of compounds developed against the HIV RNAseH and integrase that were active against the HBV RNAseH indicates that the extensive drug design efforts against these HIV enzymes can guide anti-HBV RNAseH drug discovery. Finally, differential inhibition of HBV genotype D and H RNAseHs indicates that viral genetic variability will be a factor during drug development.

## Author Summary

Current therapy for HBV blocks DNA synthesis by the viral reverse transcriptase and can control the infection indefinitely, but treatment rarely cures patients. More patients could be cured by suppressing HBV replication further using a new drug in combination with the existing ones. The HBV RNAseH is a logical drug target because it is the second of only two viral enzymes that are essential for viral replication, but it has not been exploited, primarily because it is very difficult to produce active enzyme. We expressed active recombinant HBV RNAseHs and demonstrated that it was suitable for antiviral drug screening. Twenty-one candidate HBV RNAseH inhibitors were identified based on antagonists of the HIV RNAseH and integrase enzymes. Twelve of these compounds inhibited the HBV RNAseH in enzymatic assays, and one inhibited HBV replication in cell-based assays. The high percentage of compounds developed against the HIV RNAseH and integrase that were also active against the HBV RNAseH indicates that the extensive drug design efforts against these HIV enzymes can be used to guide anti-HBV RNAseH drug discovery.

## Introduction

Hepatitis B virus (HBV) is a hepatotropic DNA virus that replicates by reverse transcription [1]. It chronically infects >350 million people world-wide and kills up to 1.2 million patients annually by inducing liver failure and liver cancer [2]–[4]. Reverse transcription is catalyzed by a virally-encoded polymerase that has two enzymatic activities: a DNA polymerase that synthesizes new DNA and a ribonuclease H (RNAseH) that destroys the viral RNA after it has been copied into DNA [1], [5]. Both activities are essential for viral replication.

HBV infections are treated with interferon α or one of five nucleos(t)ide analogs [6]–[8]. Interferon α leads to sustained clinical improvement in 20–30% of patients, but the infection is very rarely cleared [1], [3], [9]. The nucleos(t)ide analogs are used more frequently than interferon. They inhibit DNA synthesis and suppress viral replication by 4–5 log_10_ in up to 70–90% patients, often to below the standard clinical detection limit of 300–400 copies/ml [10]–[12]. However, treatment eradicates the infection as measured by loss of the viral surface antigen (HBsAg) from the serum in only 3–6% of patients even after years of therapy [10]–[13]. Antiviral resistance was a major problem with the earlier nucleos(t)ide analogs, but resistance to the newer drugs entecavir and tenofovir is very low [6], [14], [15]. This has converted hepatitis B from a steadily worsening disease into a controllable condition for most individuals [16]. The cost of this control is indefinite administration of the drugs (probably life-long; [7]), with ongoing expenses of $400–600/month [17], [18] and unpredictable adverse effects associated with decades-long exposure to the drugs.

**Figure 1:**
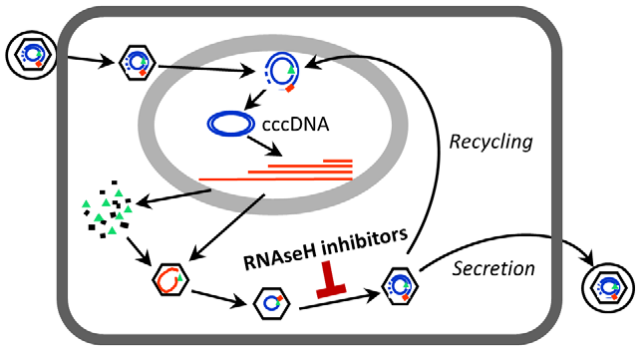
The HBV replication cycle. HBV replicates by reverse transcription in the cytoplasm of infected hepatocytes. After completion of reverse transcription, intracellular capsids can either be transported into the nucleus to maintain the cccDNA pool (*Recycling*), or they can be enveloped and secreted from the cells as mature virions (*Secretion*). Inhibiting RNAseH activity blocks plus-strand DNA synthesis during reverse transcription; this would prevent both recycling and secretion of virions. The hepatocyte is represented as a rectangle, the nucleus as an oval, HBV capsids as a hexagon, and the viral lipid envelop as a circle surrounding the extracellular capsids. HBV proteins are green or black, RNAs are red, and DNAs are blue.

The key form of the HBV genome in cells that must be eliminated to clear the infection is the nuclear episomal covalently-closed circular DNA (cccDNA) that is the template for transcription of all HBV RNAs [19]. Following reverse transcription in the cytoplasm, newly synthesized genomes can either be enveloped and secreted from the cell as virions, or they can be transported into the nucleus to replenish the cccDNA pool (Fig. 1) [19], [20]. Transfer of newly synthesized viral genomes into the nucleus via “recycling” is the default pathway, and virion secretion occurs only if the cccDNA pool is large enough to support adequate synthesis of the HBsAgs.

The cccDNA pool is very stable, but nucleos(t)ide therapy can suppress cccDNA levels in the liver by ∼1 log_10_ after 1–2 years [21]–[23]. The indefinite persistence of the cccDNA even in patients whose HBV titres in serum have been suppressed below the limit of clinical detection by the nucleos(t)ide analogs is due to residual viral replication, leading to replenishment of the cccDNA pool by a combination of intracellular recycling and low-level infection of new cells [24], [25]. The sequential accumulation of resistance mutations during nucleos(t)ide therapy confirms that cccDNA maintenance by residual viral replication occurs in the absence of clinically detectable viremia [15], [26], [27]. A recent genetic analysis of HBV DNA in the liver explicitly demonstrated that low levels of cccDNA replenishment occurs even when nucleos(t)ide analog therapy has reduced viral titres below the clinical detection limit [24].

**Figure 2:**
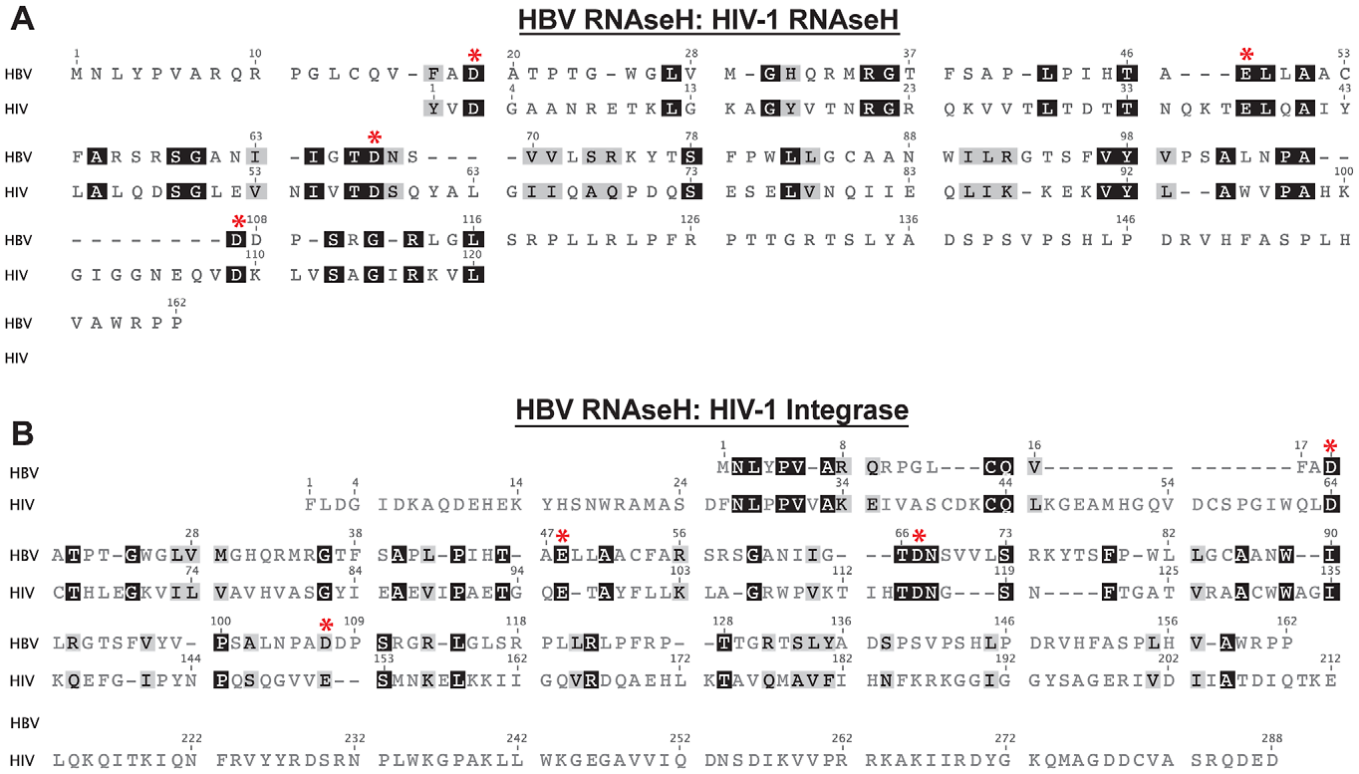
Alignments between the HBV RNAseH and the HIV-1 RNAseH and integrase. Manually optimized alignments between HBV RNAseH and **A.** the HIV-1 RNAseH, or **B.** the HIV-1 integrase. The HBV genotype D sequence is from Genbank entry V01460 and the HIV-1 sequences are from strain HXB2; Genbank K03455.1. Identical residues are shaded in black and similar residues are shaded in gray. * indicates the conserved nucleotidly transferase superfamily active site carboxylates (D-E-D-D for the RNAseH enzymes and D-D-E for the integrase). The numbering for each sequence is indicated at top. Residue 1 for the HBV RNAseH domain is amino acid 684 in the full-length polymerase protein (strain adw2) and residue 1 for the HIV-1 RNAseH domain is amino acid 441 of the full-length reverse transcriptase (strain HXB2).

RNAseH enzymes hydrolyze RNA in an RNA:DNA heteroduplex [28]. They belong to the nucleotidyl transferase superfamily whose members share a similar protein fold and presumably have similar enzymatic mechanisms [29]. This family includes *E. coli* RNAseH I and II [30], DNA transposases including the Tn5 transposase [31], retroviral integrases including the HIV integrase [32], the RuvC Holliday junction resolvase [33], the Argonaute RNAse [34], and human RNAseH 1 and 2 [35], [36]. The canonical RNAseH structure contains about 100 aa including four conserved carboxylates (the “DEDD” motif) that coordinate two divalent cations [37]. The RNAseH mechanism is believed to involve both divalent cations [29], [38], [39], although a one-ion mechanism has also been proposed [40], [41]. The HBV RNAseH domain shares low but recognizable (∼20%) sequence identity with the RNAseH domains of reverse transcriptases and other retro-elements [42]–[44]. Manually optimizing alignment of the HBV RNAseH and the HIV-1 RNAseH yielded 23% identity and 33% similarity (Fig. 2). A similar alignment between the HBV RNAseH and the HIV integrase revealed 19% identity and 33% similarity.

The HBV RNAseH is encoded at the carboxy-terminus of the viral polymerase protein that also encodes the viral DNA polymerase activity (reverse transcriptase). The high hydrophobicity of the HBV polymerase and its existence as a complex with host chaperones [45] have severely restricted study of the HBV RNAseH. Furthermore, we demonstrated that the RNAseH in its native context within the polymerase protein is unable to accept exogenous heteroduplex substrates [46], analogous to the inability of the DNA polymerase active site to engage exogenous primer-templates [47]. Consequently, most of our limited knowledge of the RNAseH comes from mutational studies of the viral genome in the context of viral replication conducted by us and others [48]–[53]. These restrictions have prevented biochemical characterization of the RNAseH and blocked biochemical screens for anti-HBV RNAseH drugs to date.

A few reports of recombinant forms of the hepadnaviral RNAseH exist. Wei and co-workers [54] expressed the HBV RNAseH domain in *E. coli* and purified it by denaturing nickel-affinity chromatography. Following refolding, they found an RNAse activity. Lee et al. [55] expressed the HBV RNAseH domain in *E. coli* as a dual maltose-binding protein/hexahistidine fusion and purified soluble protein by two-step affinity chromatography; this enzyme had RNAseH activity. Choi and co-workers [56] expressed the intact duck hepatitis B virus polymerase in yeast and reported that it had a weak RNAse activity. Finally, Potenza et al. [57] expressed the HBV RNAseH domain as a synthetic gene in *E. coli*. Following purification from inclusion bodies and refolding, this enzyme had RNAse activity. However, no follow-up reports have appeared with any of these systems, possibly due to the technical difficulties associated with the purification protocols and/or contamination challenges with host RNAseH or other RNAse classes.

Human Immunodeficiency Virus (HIV) reverse transcription also requires a virally encoded RNAseH activity [58], and consequently the RNAseH has attracted much attention as a potential drug target [38], [59]–[78]. Over 100 anti-HIV RNAseH compounds have been reported, typically with inhibitory concentration-50% (IC_50_) values in the low µM range. Most of the compounds inhibit HIV replication in culture, typically with effective concentration-50% (EC_50_) values that are ∼10-fold higher than the biochemical IC_50_ values. These compounds are often modestly cytotoxic, leading to therapeutic indices (TI) that are usually <10. Second-generation inhibitors with substantially improved efficacy have been reported, but their TI values were not necessarily improved markedly [68]–[70]. Despite these limitations, compounds with efficacy and TI values appropriate for a drug exist [68], [75]. Most of the compounds inhibit the RNAseH by binding to the enzyme and chelating the divalent cations in the active site [64], [65], [69], [70], [73], [76], but compounds that appear to inhibit the RNAseH by altering the enzyme's conformation or its interaction with nucleic acids have also been reported [63], [75]. As predicted from their common membership in the nucleotidyl transferase superfamily, some anti-HIV RNAseH compounds can inhibit the HIV integrase, and some anti-integrase compounds can inhibit the RNAseH [59], [68], [70], [74], [79].

The ability of the nucleos(t)ide analog drugs to profoundly suppress HBV in most patients and to cure HBV infection in a few patients indicates that they can push the virus to the brink of elimination. This presents an opportunity to cure many more patients by suppressing HBV replication further, but achieving a cure will require novel drugs against targets other than the DNA polymerase active site. These drugs would be used in combination with the nucleos(t)ide analogs to suppress viral replication below the level needed to maintain the cccDNA. A logical target would be the second of HBV's two enzymatic activities, the RNAseH. Here, we report production of enzymatically active recombinant HBV RNAseH suitable for low throughput antiviral drug screening. Using this novel reagent, we demonstrated that the HIV RNAseH and integrase are similar enough to the HBV RNAseH to allow information derived from HIV RNAseH and integrase inhibitors to guide identification of anti-HBV RNAseH compounds.

## Results

### Confirmation of key HBV RNAseH active site residues

**Figure 3:**
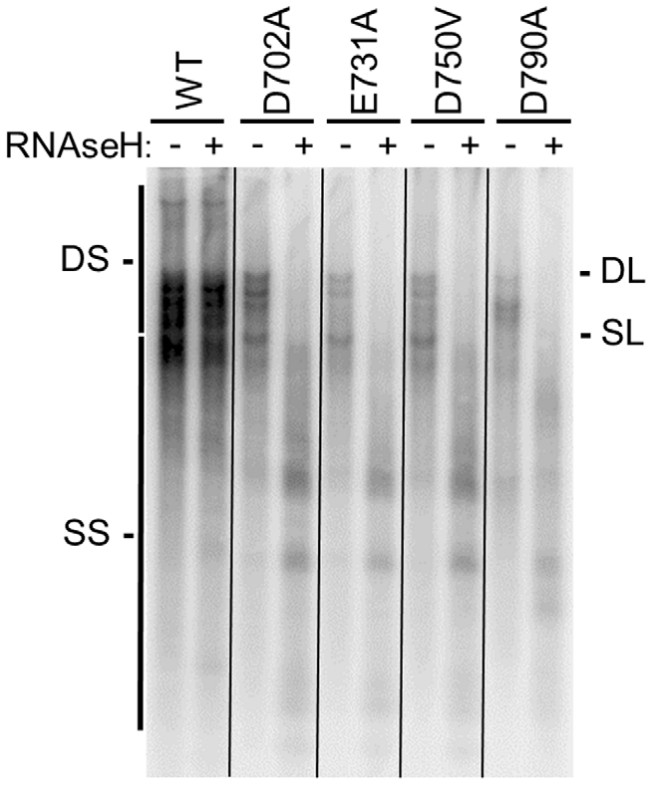
Identification of the DEDD motif in the HBV RNAseH active site. Wild-type and mutant HBV genotype A genomic expression vectors were transfected into cells, intracellular capsids were isolated five days later, and viral nucleic acids were purified from the capsids. The nucleic acids were divided into two aliquots; one aliquot was treated with DNAse-free *E. coli* RNAseH to destroy RNA:DNA heteroduplexes and the other was mock treated. The nucleic acids were resolved by agarose electrophoresis and HBV DNAs were detected by Southern analysis. The signature of an RNAseH-deficient genome is production of RNA:DNA heteroduplexes in which the DNA migrates as double-stranded species without treatment with exogenous RNAseH treatment but as singe-stranded species following degradation of the RNA. The positions of the duplex linear (DL) and full-length single-stranded linear (SL) HBV DNA markers are shown. DS indicates the spectrum of double-stranded nucleic acids produced by reverse transcription, and SS indicates the spectrum of single-stranded nucleic acids.

The HBV DEDD residues have been implicated to be D702, E731, D750, and D790 (numbering for HBV strain adw2) by sequence alignments against other RNAseHs (Fig. 2), but only D750 has been experimentally confirmed to be essential for RNAseH activity [48]. Therefore, we introduced D702A, E731A, D750V, and D790A mutations into the predicted DEDD motif residue in an HBV genomic expression vector. The wild-type and mutant genomes were transfected into Huh7 cells, five days later intracellular viral capsids were purified, and then HBV DNAs within the particles were detected by Southern analysis. All four mutants supported DNA synthesis and hence could be analyzed by this approach. The signature of an RNAseH-deficient enzyme is production of RNA:DNA heteroduplexes that migrate like double-stranded DNAs on native gels but as faster-migrating single-stranded DNAs of multiple lengths following digestion of the capsid-derived nucleic acids with exogenous RNAseH. DNAs produced by the wild-type genome were unaffected by treatment with RNAseH prior to electrophoresis (Fig. 3). Mutating each of the four predicted RNAseH DEDD residues blocked production of the slowest-migrating double stranded forms (mature relaxed-circular DNAs) and led to accumulation of smaller forms that migrated similar to the less-mature relaxed-circular DNAs produced by the wild-type genome. Treatment of the nucleic acids from the mutant genomes with exogenous RNAseH collapsed the double-stranded forms to single-stranded forms (Fig. 3). Therefore, all four mutants were RNAseH deficient.

### Production of enzymatically active recombinant HBV RNAseH

**Figure 4:**
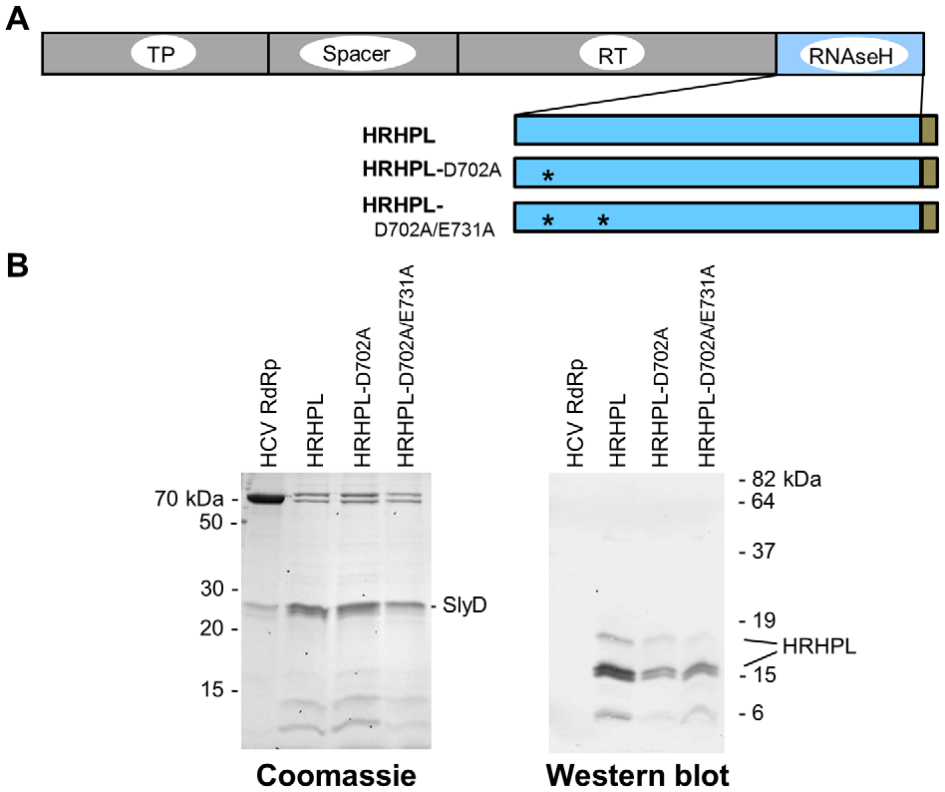
Recombinant HBV RNAseH proteins. **A.** Structure of the recombinant RNAseHs. The HBV polymerase with its major domains labeled is at top. The recombinant RNAseH derivatives are shown below with the C-terminal hexahistidine tag in brown. TP, terminal protein domain; RT, reverse transcriptase domain; *, mutations D702A or E731A to RNAseH active site residues. **B.** Proteins in the enriched lysates. The left panel is a Coomassie-blue stained SDS-PAGE gel of enriched RNAseH extracts as employed in the RNAseH assays. The right panel is a western blot of the extracts employing monoclonal antibody 9F9 which recognizes an epitope near the C-terminus of the HBV polymerase.

We expressed HBV RNAseH sequences from the HBV isolate employed by Potenza et al. [57] in *E. coli* as a carboxy-terminally hexahistidine tagged recombinant protein, but we moved the amino terminus nine residues upstream to residue 684 of the HBV polymerase because we felt this site was more probable to yield soluble protein (HRHPL; Fig. 4A). As a negative control, we mutated two of the DEDD active site residues (D702A and E731A). These constructs were expressed in *E. coli*, soluble lysates were prepared, and the lysates were subjected to nickel-affinity chromatography.

Five proteins of approximately 80, 70, 26, 14, and 11 kDa detectable by Coomassie staining were recovered following chromatography, none of which correlated with the predicted mass of 18.9 kDa for HRHPL (Fig. 4B). Mass spectrometry identified the dominant 26 kDa band as the *E. coli* prolyl isomerase SlyD. Concentrating the samples seven-fold did not increase the RNAseH to levels detectable by Coomassie staining. Western analysis with anti-polyhistidine antibodies revealed a large number of cellular bands but failed to unambiguously identify HRHPL. This was presumably due to the presence of histidine-rich regions in the bacterial proteins that promoted their binding to the nickel-affinity resin (e.g., SlyD). However, western analysis with the anti-HBV RNAseH domain antibody 9F9 ([80]; Santa Cruz Biotechnology) revealed a small amount of recombinant HBV RNAseH that migrated close to its predicted mass plus a larger amount of the protein that migrated as a doublet near 15 kDa (Fig. 4B). The doublet is presumably due to proteolysis near the protein's N-terminus because the antibody epitope and hexahistidine tag are at the C-terminus. The sizes of the truncation products imply that they were cleaved near HRHPL residue 36, which would remove the essential D702 carboxylate (HRHPL residue 20) and inactivate the protein. These experiments indicate we could express and enrich small but detectable amounts of soluble recombinant HBV RNAseH.

**Figure 5:**
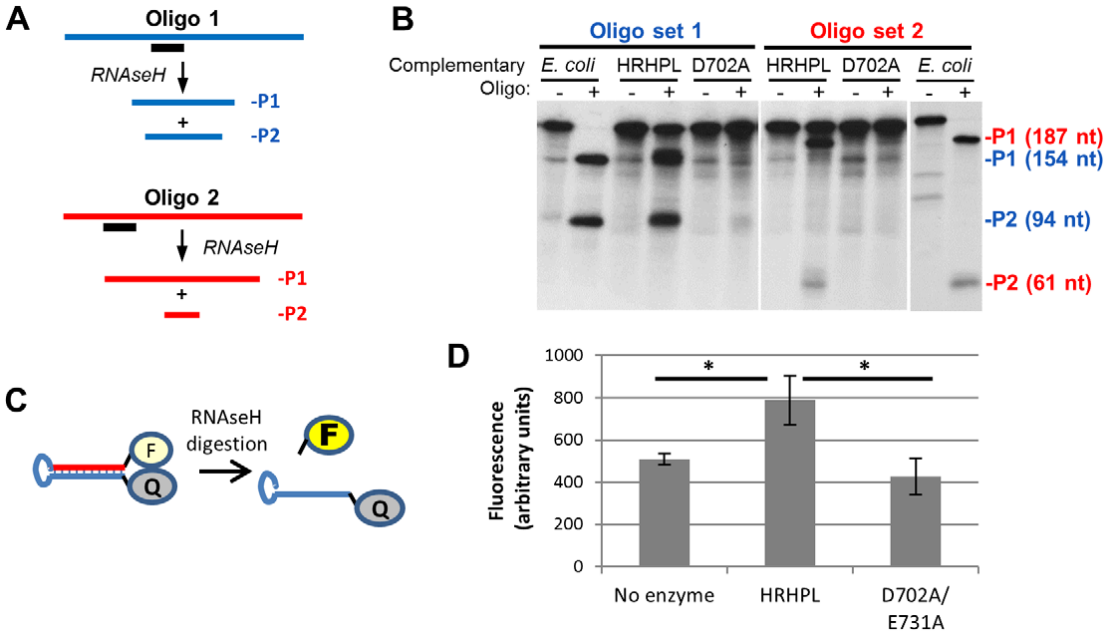
Recombinant HBV RNAseH is enzymatically active. **A.** Oligonucleotide-directed RNAseH assay. Uniformly ^32^P-labeled RNA (blue or red) is annealed to a complementary DNA oligonucleotide (black). RNAseH activity cleaves the RNA in the heteroduplex formed where the oligonucleotide anneals to the RNA and yields two products (P1 and P2). **B.** Recombinant HBV RNAseH is active. An oligonucleotide-directed RNAseH assay was conducted with *E. coli* RNAseH, wild-type HBV RNAseH (HRHPL), or RNAseH-deficient HRHPL (D702A). A complementary oligonucleotide (+) or non-complementary oligonucleotide (−) was mixed with labeled DRF+ RNA and the reactions were incubated to allow RNAseH activity. The products were resolved by SDS-PAGE and the RNAs were detected by autoradiography. Oligonucleotide set 1 was D2507− and D2526+ and oligonucleotide set #2 was D2543M-Sal and D2453+. The positions of the cleavage products (P1 and P2) are indicated in blue for reactions containing oligonucleotide D2507− and in red for reactions containing oligonucleotide D2543M-Sal. **C.** FRET-based RNAseH assay. A self-complementary chimeric RNA:DNA synthetic oligonucleotide (RHF1) forms a stem-loop in which the stem is an RNA:DNA heteroduplex. The stem brings the fluorescein (F) and quencher (Q) at the 5′ and 3′ ends of the oligonucleotide into close proximity. Cleavage of the RNA releases the fluorescein and increases its fluorescence. **D.** Detection of HBV RNAseH activity employing the fluorescent assay. The substrate in panel C was employed in an RNAseH assay employing buffer alone, wild-type HBV RNAseH (HRHPL), or RNAseH-deficient HRHPL (D702A/E731A). *, P<0.05.

We tested activity of the recombinant HBV RNAseHs in a DNA oligonucleotide-directed RNA cleavage assay. In this assay, a DNA oligonucleotide is annealed to a uniformly-labeled RNA to create an RNA:DNA heteroduplex. Cleavage of the RNA in the heteroduplex yields two RNA fragments of predictable size that are resolved by electrophoresis and detected by autoradiography (Fig. 5A). We employed the 264 nt RNA (DRF+) used in our previous RNAseH assays [46] in combination with two DNA oligonucleotide pairs. One oligonucleotide in each pair was the correct polarity to anneal to the DRF+ RNA and the other was its inverse complement as a negative control.

**Figure 6:**
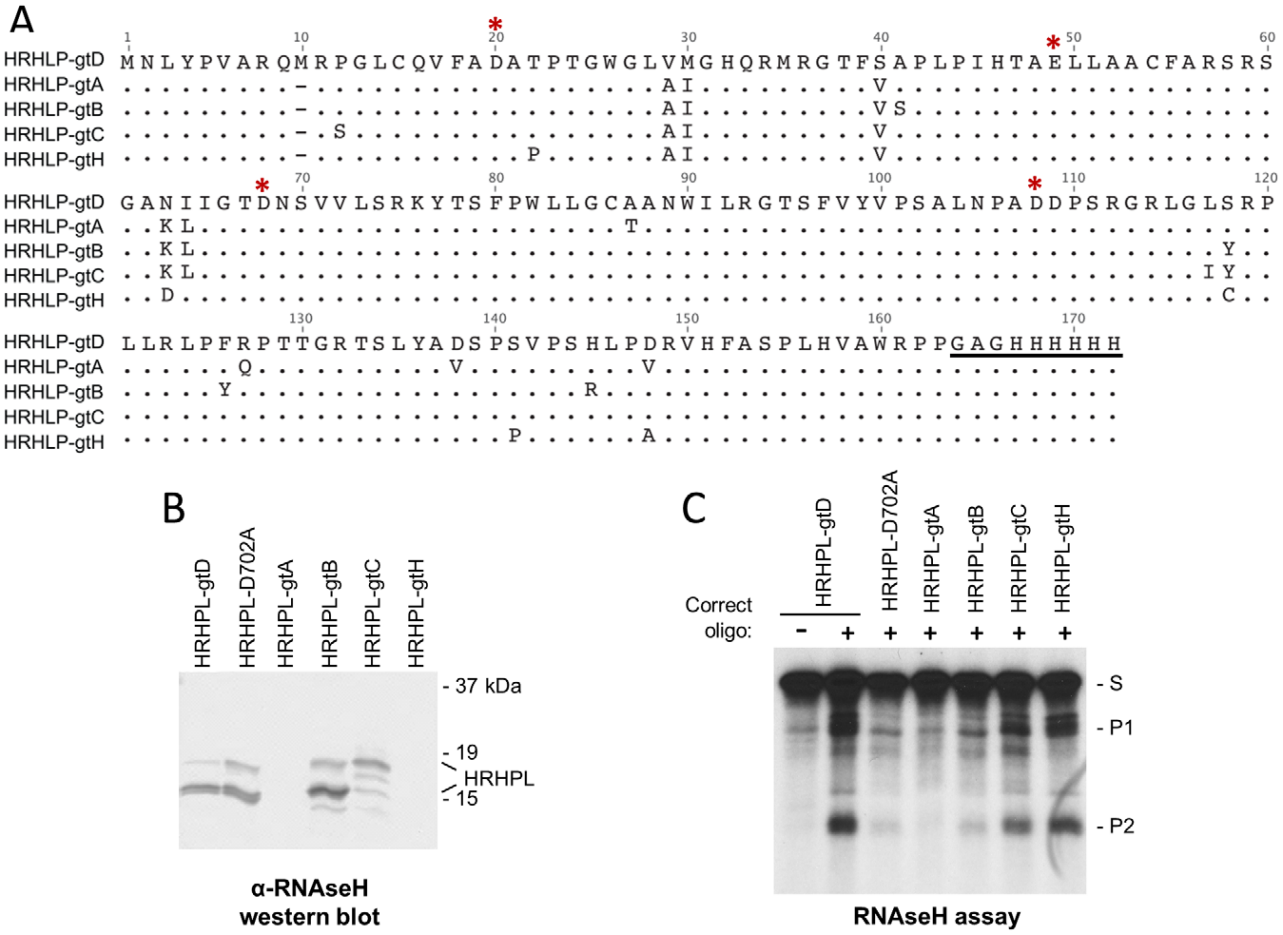
Recombinant RNAseHs from HBV genotypes A, B, C, D, and H. **A.** Sequence alignment for genotype A, B, C, D, and H versions of the HBV RNAseH expression construct HRHPL. The additional methionine at residue 10 of the genotype D sequence is a product of the cloning strategy; this insertion has no impact on the RNAseH activity because the first 9 amino acids of HRHPL can be deleted without altering the biochemical profile of the enzyme. * indicates the DEDD active site residues, and the hexahistidine tag at the C-terminus is underlined. Residue 1 for the HBV RNAseH domain is amino acid 684 in the full-length polymerase protein (strain adw2). **B.** Western analysis of RNAseH proteins in the enriched lysates probed with the anti-HBV RNAseH monoclonal antibody 9F9. **C.** RNAseH activity of RNAseH from HBV genotypes A, B, C, D, and H detected by the oligonucleotide-directed RNA cleavage assay. HRHPL-D702A (genotype D) is a negative control. gt, genotype.

Oligonucleotide-directed RNAseH assays were conducted with wild-type HRHPL enzyme and the RNAseH-deficient D702A mutant. The RNA was not cleaved when the non-complementary oligonucleotides were employed in the reactions (Fig. 5B), demonstrating that the enzyme preparations did not contain non-specific RNAse activity. Use of complementary oligonucleotide #1 (D2507−) led to complete cleavage of the DRF+ RNA by *E. coli* RNAseH into products of 154 and 94 nt, and to partial cleavage of the RNA at the same site by wild-type HRHPL (Fig. 5B). The large majority of this RNAseH activity was due to the HBV enzyme because mutating DEDD residues D702A and/or E731A sharply reduced cleavage of the RNA. Note that although the relative yield of full-length mutant RNAseH was less than the wild-type enzyme in Fig. 4, in other preparations the amount of mutant RNAseH exceeded the amount of wild-type enzyme (e.g., Fig. 6). In all cases, the enzymatic activity associated with the mutant RNAseH preparations was far lower than in the wild-type preparations. The residual cleavage products in reactions with the mutant enzymes appear to be non-specific breakdown products from the RNA substrate and/or digestion products from trace contamination with bacterial RNAseH. The RNA products shifted sizes as expected when complementary oligonucleotide #2 (D2543M-Sal, which anneals 33 nt closer to the 3′ end of the RNA) was employed in the RNAseH assays (Fig. 5B): the larger fragment became larger (187 nt) and the smaller fragment became smaller (61 nt). These data demonstrate that the RNAse activity in HRHP is specific for RNA annealed to the DNA oligonucleotides, and hence confirm that it is an RNAseH activity.

Finally, we synthesized a quenched fluorescent RNA:DNA chimeric hairpin oligonucleotide substrate (RHF1) to confirm RNAseH activity with a different assay. RHF1 has fluorescein at its 5′ end, 20 nt of RNA, a 4 nt DNA hairpin, 20 nt of DNA complementary to the RNA, and an Iowa Black FQ quencher at the 3′ terminus. The hairpin brings the fluorescein and quencher into close proximity, and digesting the RNA frees the fluorescein and increases its fluorescence (Fig. 5C). RHF1 was terminally digested with *E. coli* RNAseH, the reactions were terminated with 10 mM EDTA, and fluorescence was measured. This digestion amplified the fluorescence of RHF1 22-fold, indicating a 95% quenching efficiency. RHF1 was then employed in an RNAseH assay with buffer alone, wild type HBV RNAseH (HRHPL), and HRHPL-D702A/E731A. RNAseH activity for HRHPL was about 2-fold higher than the no-enzyme control, and mutating the RNAseH active site eliminated this activity (Fig. 5D). This weak signal (7% of the maximal signal in this assay) appears to be due to poor binding between the small substrate and the RNAseH in the relatively high ionic strength of the reactions because detection of RNAseH activity required reducing the NaCl concentration from 190 to 130 mM.

These data indicate that we can readily detect HBV RNAseH activity in the enriched bacterial extracts despite the fact that the HBV RNAseH is a minor component of the mixture.

### Optimization of reaction conditions

The optimal enzymatic conditions for the HRHPL HBV RNAseH were determined by systematically varying the reaction components in the oligonucleotide-directed RNAseH assay (Table 1). Recombinant HBV RNAseH was active over a wide range of pH values but was most active near 8.0. Its activity maximum was at 190 mM NaCl and it became able to digest single-stranded RNA below ∼100 mM NaCl. The RNAseH required ∼5 mM Mg^++^ for maximal activity; increasing Mg^++^ beyond ∼7 mM suppressed RNAseH activity, and inclusion of Mn^++^ in the reactions led to nonspecific degradation of single-stranded RNA. The enzyme became inactive at low reductant concentrations, but it could tolerate up to 2% DMSO. It was stable upon storage in liquid nitrogen, and only marginal loss of activity was observed following five sequential freeze-thaw cycles.

**Table 1:** Optimal reaction conditions.

Tris pH 7.5	65 mM
NaCl	190 mM
MgCl_2_	5 mM
DTT	5 mM
Glycerol	6%
DMSO	1%
NP40	0.05%
DNA Oligo (20 mer)	0.15 µg/µl
RNA (264 nt)	0.025 µg/µl
Temperature	42°C
RNAseIn	0.5 U/µl

### Recombinant RNAseH enzymes from other HBV genotypes

HBV has eight genotypes (A–H, plus provisional identification of genotypes I and J) that differ by >8% at the sequence level [81]. We cloned HBV RNAseH domains for genotype A, B, C, and H isolates using the same structure as the HRHPL construct (genotype D) to determine whether HBV's genetic diversity leads to variable sensitivity to inhibitors that must be taken into account during drug development (Fig. 6A). The protein profile detectable by Coomassie staining following expression and nickel-affinity enrichment for all additional constructs was the same as for HRHPL. Western blotting with antibody 9F9 detected the genotype B, C, and D RNAseHs, with the genotype C enzyme appearing primarily as the full-length protein (Fig. 6B). The failure to detect the genotype A and H RNAseHs was due either to lack of accumulation of the proteins or to amino acid variations in the C-terminus of the protein where the antibody epitope is located [80].

The genotype A, B, C, D, and H RNAseH extracts were assessed with the oligonucleotide-directed RNAseH assay (Fig. 6C). The genotype A and B enzymes were inactive, activity of the genotype C RNAseH ranged from undetectable to modest in replicate experiments, and activity of the genotype H enzyme was similar to that of the genotype D RNAseH. The [NaCl]-, [Mg^++^]-, temperature-, and pH-profiles of the genotype H RNAseH were very similar to those of the genotype D enzyme (data not shown).

Therefore, we can express recombinant HBV genotype B, C, D, and H RNAseH proteins that are detectable by enzymatic assays and/or western blotting, but only the genotype D and H proteins are consistently active.

### Identification of anti-HBV RNAseH compounds

**Figure 7:**
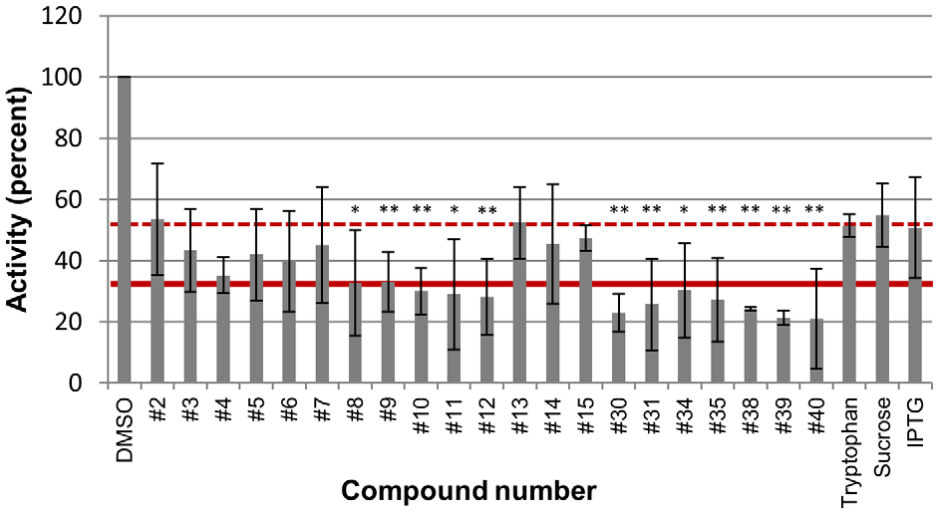
Inhibition of the HBV RNAseH by candidate compounds selected for their similarity to antagonists of the HIV RNAseH and integrase. Candidate inhibitors (compounds #2-40) and irrelevant compounds (tryptophan, sucrose, and IPTG) were included at 10 µM in a standard oligonucleotide-directed RNAseH assay employing wild-type genotype D HBV RNAseH (HRHPL). DMSO, vehicle control. Error bars are ± one standard deviation from three to seven replicates. The dashed red line indicates the mean residual activity in the irrelevant control reactions (52%) and the solid red line is two standard deviations of the irrelevant control assays below their mean (33%). Compounds that inhibited the RNAseH to 33% or below were considered to be positive (“+” in Table 2). *, P<0.05 by T-test against the pooled data for the irrelevant controls; **, P<0.01.

**Figure 8:**
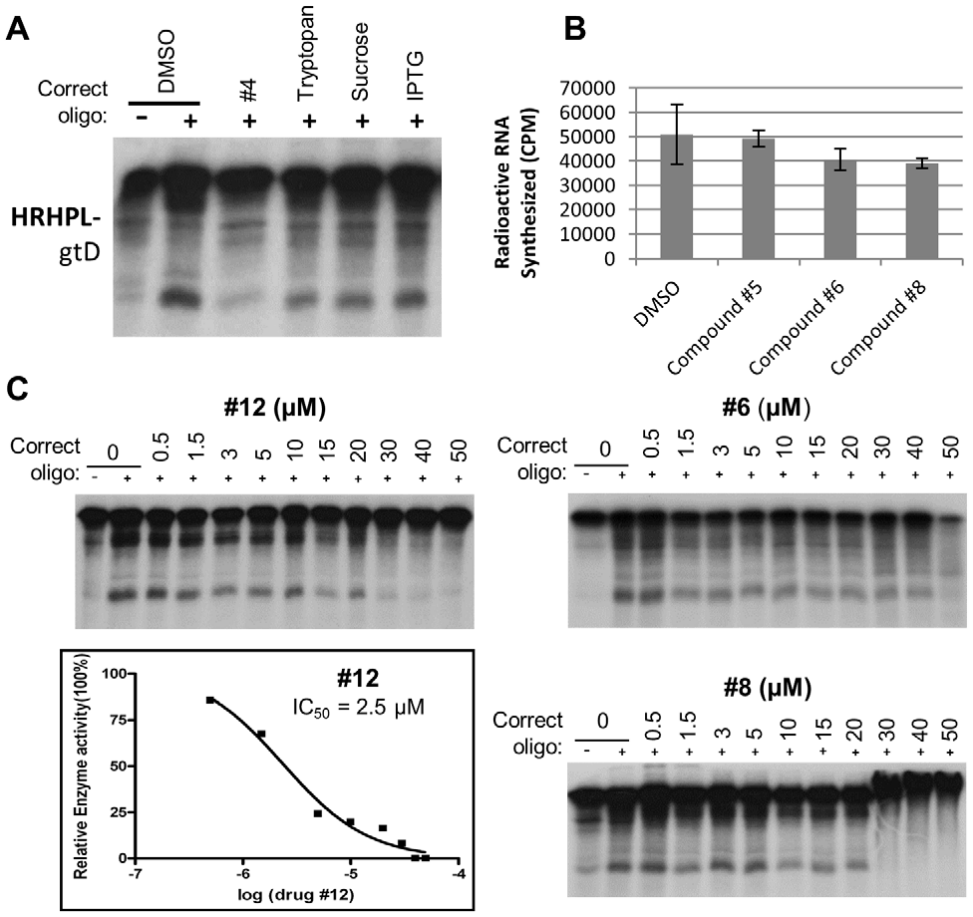
Specificity of anti-HBV RNAseH compounds. **A.** Inhibition of HBV genotype D RNAseH by irrelevant compounds at 10 µM in the oligonucleotide-directed RNAseH assay. Compound #4 was employed as an example HBV RNAseH inhibitor. **B.** Anti-HBV RNAseH inhibitors do not significantly inhibit the HCV RNA polymerase. The ability of compounds #5, 6 and 8 to inhibit production of poly-G by the HCV RNA-directed RNA polymerase was measured in a primed homopolymeric RNA synthesis assay [82]. The compounds were employed at 10 µM. DMSO, vehicle control. **C.** Dose-responsiveness of HBV RNAseH inhibition. The effects of compounds #6, 8, and 12 on the RNAseH activity of HRHPL (genotype D) were measured at concentrations ranging from 0.5 to 50 µM. The dose-response profile is plotted for compound #12.

We hypothesized that the HBV RNAseH may be inhibited by antagonists of the HIV RNAseH based on the similarity of the reactions they catalyze. We identified 10 compounds known to inhibit the HIV RNAseH or that were predicted by chemical structure-activity relationships to do so (Table 2 and Supplementary Fig. S1). We further hypothesized that anti-HIV integrase compounds may inhibit the HBV RNAseH because the integrase and RNAseH are both members of the nucleotidyl transferase superfamily and because some anti-HIV RNAseH and integrase compounds can cross-inhibit their target enzymes [59], [68], [70], [74], [79]. Consequently, we also obtained 11 compounds either known to inhibit the HIV integrase or predicted to do so by chemical structure-activity relationships (Table 2 and Supplementary Fig. S1). We first measured the effect of irrelevant compounds (tryptophan, sucrose, and IPTG) on the RNAseH assay. These compounds reduced RNAseH activity of HRHPL to 52±9% relative to the DMSO vehicle control (Figs. 7 and 8A). This allowed us to define the mean of the residual activity in the presence of the irrelevant compounds minus two standard deviations of the irrelevant controls as a threshold reduction of the RNAseH activity that must be exceeded before we considered inhibition by the test compounds to be relevant. Using this threshold, 12 of the 21 compounds inhibited the HBV genotype D RNAseH at 10 µM (Fig. 7, Table 2, and Supplementary Table S1). These 21 compounds were also screened against the HBV genotype H RNAseH using the oligonucleotide-directed RNAseH assay.

**Table 2:** Candidate RNAseH inhibitors and data summary.

Compound			RNAseH assays^2^		HBV Replication^3^	
Number	Name^1^	HIV template	gtD	gtH	gtA	gtD
2	Sigma 74540	RNAseH	**−**	**−**	**−**	**−**
3	Sigma n8164	RNAseH	**−**	**−**		**−**
4	TimTec ST029023	RNAseH	**+/−**	**−**	**−**	**−**
5	Enamine T0506-3483	RNAseH	**+/−**	**−**	**−**	**−**
6	Chembridge 7929959	RNAseH	**+/−**	**−**	**−**	**−**
7	Idofine 02030	Integrase	**−**	**−**		**−**
8	Sigma S439274	Integrase	**+**	**−**	**−**	**−**
9	Sigma 70050	Integrase	**+**	**−**		**−**
10	Selleck S2001	Integrase	**+**	**−**		**−**
11	Selleck S2005	Integrase	**+**	**−**		**−**
12	Napthyridinone^4^	RNAseH	**+**	**++**	**+**	**+**
13	DHBNH^4^	RNAseH	**−**	**+/−**	**−**	
14	THBNH^4^	RNAseH	**−**	**+**	**+/−**	**−**
15	BHMP07^4^	RNAseH	**−**	**+/−**	**−**	
30	Chembridge 7248520	Integrase	**++**	**+/−**	**−**	
31	Chembridge 5104346	Integrase	**+**	**−**	**−**	
34	Indofine D-009	Integrase	**+**	**+**	**−**	
35	TCI America D1118	Integrase	**+**	**+/−**		**−**
38	Vistas M Lab STK317995	RNAseH	**++**	**+/−**		**−**
39	Asinex BAS0223612	Integrase	**++**	**+**		**−**
40	NIH 118-D-24	Integrase	**++**	**++**	**+/−**	**−**

1 Structures are in Supplemental Fig. S1.

2 Inhibitory activity in the biochemical RNAseH assay at 10 µM; quantitative data are in Supplemental Table S1. Cutoff values were established relative to the mean and standard deviation of RNAseH activity in the presence of the irrelevant compounds tryptophan, sucrose, and IPTG normalized to the vehicle control. −, Residual activity greater than one standard deviation below the mean irrelevant inhibition (≥43% activity); +/−, Residual activity between one and two standard deviations below the mean irrelevant inhibition (42–34% activity); +, residual activity between two and three standard deviations below the mean irrelevant inhibition (33–25% activity); ++, residual activity less than three standard deviations below the mean irrelevant inhibition (≤24% activity).

3 Inhibitory activity against HBV replication in Huh7 cells at 10 µM. +, clear inhibition; −, no detectable inhibition; +/−, inhibition observed in some but not most assays.

4 Napthyridinone [68]; DHBNH, dihydroxy benzoyl naphthyl hydrazone [75], [78]; THBNH, trihydroxy benzoyl naphthyl hydrazone [106]; BHMP07 [78].

The unexpectedly high frequency of inhibition of the genotype D enzyme led us to question the mechanism(s) by which it was inhibited by the compounds. We addressed this in two manners. First, RNAseH inhibitors usually block the HIV enzyme by interfering with the divalent cations in the active site [64], [65], [69], [70], [73], [76]. Consequently, we asked whether the compounds act non-specifically by chelating Mg^++^. Isothermal calorimetry demonstrated that compounds #5, 6, and 8 did not bind Mg^++^ in the absence of the protein extracts (data not shown). This is consistent with their inability to significantly inhibit poly-G synthesis by the Hepatitis C virus (HCV) RNA polymerase which is also active in 5 mM Mg^++^ [82] (Fig. 8B). Second, we titrated selected compounds from 50 to 0.5 µM to examine dose-responsiveness of inhibition (Fig. 8C). Compound #12 had a typical inhibition curve with an IC_50_ of 2.5 µM in this experiment; similar smooth dose-response curves were observed for compounds #39 and 40 (data not shown). In contrast, inhibition by compound #6 plateaued at 20–30% between 3 and 40 µM but then increased to 75% at 50 µM. Compound #8 was ineffective below 5 µM, it inhibited the enzyme by 40–85% between 10 and 30 µM, and caused aberrant migration of the RNA at 40 and 50 µM. These data indicate that some compounds behaved as predicted from their mechanism of action against HIV, but that inhibition by other compounds may have been due to alternative effects, possibly including interaction with the RNA and/or aggregation of the enzyme.

### Activity of HBV RNAseH inhibitors against human RNAseH1

**Figure 9:**
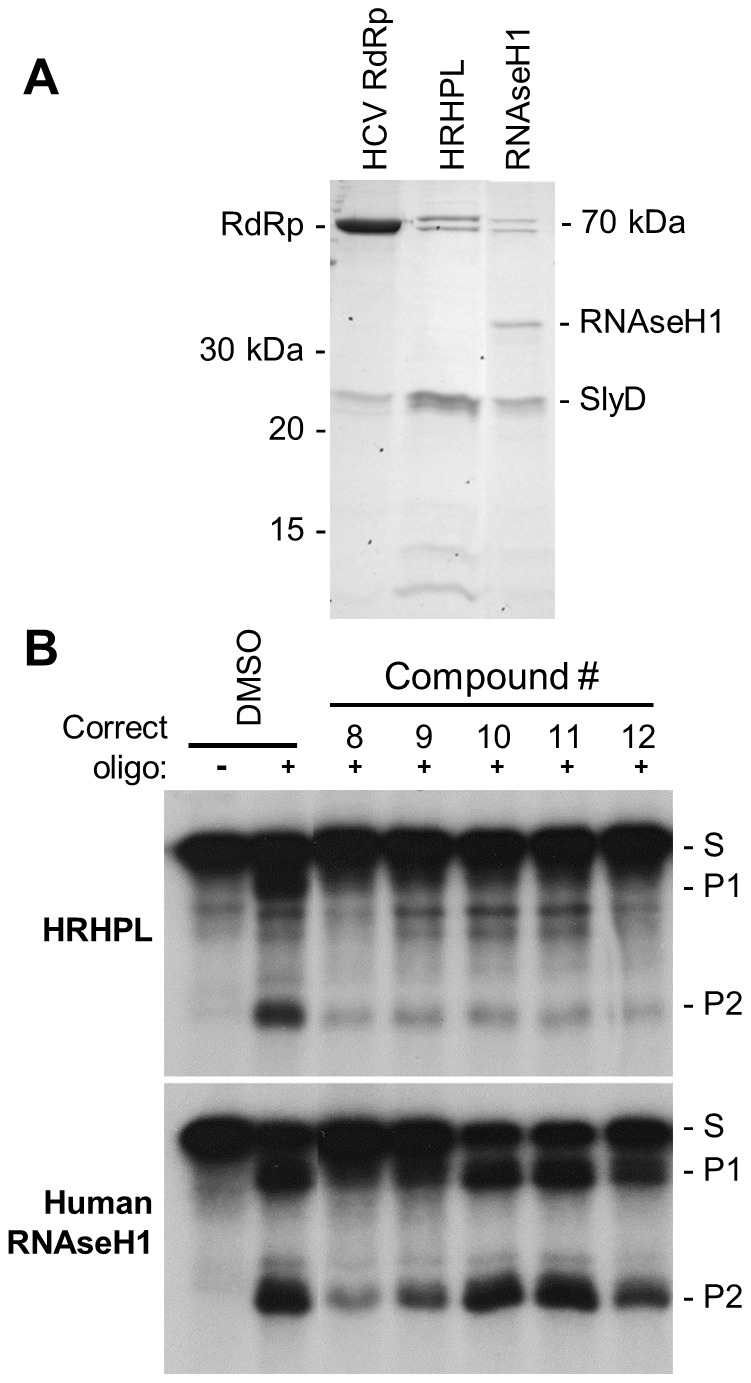
Activity of HBV RNAseH inhibitors against human RNAseH1. **A.** Proteins in the enriched recombinant human RNAseH1 lysates employed in the RNAseH reactions were detected by Coomassie-blue staining following SDS-PAGE. **B.** An oligonucleotide-directed RNAseH assay was conducted with wild-type HBV RNAseH (genotype D) and recombinant human RNAseH1 under identical reaction conditions. The inhibitory compounds were employed at 10 µM. The upper and lower panels are from the same experiment and the data were collected on a single sheet of film, so the reactions can be directly compared. DMSO, vehicle control. S, the DRF+ substrate; P1 and P2, RNAseH cleavage products.

A likely cause of cellular toxicity for anti-HBV RNAseH drugs would be inhibition of human RNAseH1 because it is responsible for about 80% of the RNAseH activity in human cells [83], [84]. Therefore, we cloned the human RNAseH1 with an N-terminal hexahistidine tag, expressed it in *E. coli*, and enriched the protein by nickel affinity chromatography. The same spectrum of contaminating *E. coli* proteins as was observed for the other RNAseH preparations was detectable by Coomassie staining, but RNAseH1 could be detected at its predicted mass of 32 kDa (Fig. 9A). This enzyme was active in the oligonucleotide-directed and fluorescent RNAseH assays (Fig. 9B and data not shown). To determine how inhibition of human RNAseH1 compared to inhibition of the HBV RNAseH, we titrated RNAaseH1 to yield similar levels of activity as the HBV enzyme, and then we directly compared the ability of compounds #8-12 to inhibit human RNAseH1 and HRHPL at 10 µM. All five compounds inhibited the HBV RNAseH. Compound #8 inhibited RNAseH1 well, #9 and 12 inhibited it weakly, and #10 and 11 had no effect on RNAseH1. Therefore, it is possible to inhibit the HBV RNAseH without inhibiting human RNAseH1.

### Anti-HBV RNAseH compounds can inhibit HBV replication in culture

Finally, we asked whether HBV RNAseH inhibitors could block HBV replication in culture. Huh7 cells were transfected with genomic expression vectors for HBV genotype A or D isolates, the cells were treated with 10 or 50 µM compounds, and viral nucleic acids were isolated from intracellular HBV capsids after four days. Replicate nucleic acid aliquots were mock treated or treated with DNAse-free *E. coli* RNAseH to destroy RNA:DNA heteroduplexes, and then HBV DNAs were detected by Southern blotting. The signature of RNAseH inhibition is accumulation of RNA:DNA heteroduplexes that migrate as double-stranded species without exogenous RNAseH treatment but as faster-migrating single-stranded DNAs following RNAseH treatment.

**Figure 10:**
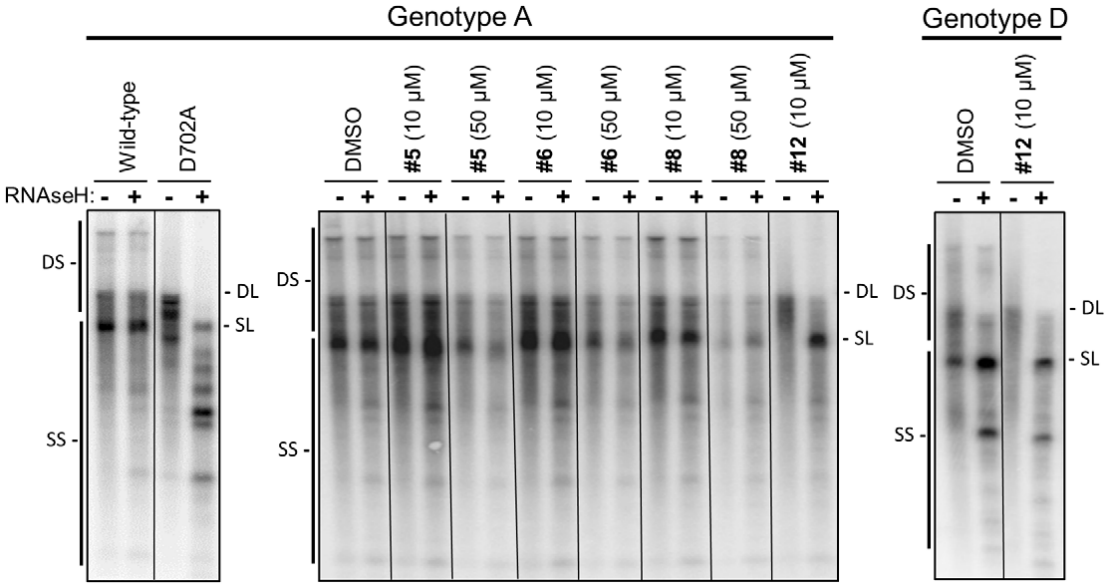
Inhibition of HBV replication in culture by RNAseH inhibitors. Genotype A or D HBV genomic expression vectors were transfected into cells, intracellular capsids were isolated four days later, and viral nucleic acids were purified from the capsids. The nucleic acids were divided into two aliquots; one aliquot was treated with DNAse-free *E. coli* RNAseH to destroy RNA:DNA heteroduplexes and the other was mock treated. The nucleic acids were resolved by agarose electrophoresis and HBV DNAs were detected by Southern analysis. Inhibition of RNAseH activity leads to accumulation of RNA:DNA heteroduplexes in which the DNA migrates as double-stranded species in the mock-treated sample but as faster-migrating singe-stranded species following RNAseH treatment. The left panel is a control in which wild-type and RNAseH-deficient D702A HBV genomes were compared. The right two panels employed wild-type HBV in the presence of the test compounds. Compounds were employed at 10 or 50 µM as indicated. DMSO, vehicle control. The positions of the duplex linear (DL) and full-length single-stranded linear (SL) HBV DNA markers are shown. DS indicates the spectrum of double-stranded nucleic acids produced by reverse transcription, and SS indicates the spectrum of single-stranded nucleic acids.

The mobility of the DNAs synthesized in cells containing the wild-type genotype A genome was unaffected by exogenous RNAseH treatment (Fig. 10). Ablation of RNAseH activity by the D702A mutant altered migration of the double-stranded forms, and treatment of these samples with RNAseH collapsed the double-stranded forms to single-stranded DNAs (Fig. 10 left panel). The mobility of HBV DNAs from cells replicating HBV genotype A treated with DMSO was unaffected by RNAseH digestion (Fig. 10 center panel), but treatment of cells with compound #12 at 10 µM blocked production of the slowest-migrating double-stranded forms and led to accumulation of RNA:DNA heteroduplexes whose mobility increased upon removal of RNA. Treatment of cells with 3 to 50 µM compound #12 revealed that the degree of inhibition was proportional to the concentration of the compound (data not shown). Plus-strand preferential real-time PCR across the gap in the minus-polarity viral DNA revealed that 10 µM compound #12 reduced plus-strand DNA accumulation to 7.3% of the DMSO-treated control (data not shown). None of the other compounds reproducibly inhibited HBV genome synthesis (Table 2), but compound #14 (25 µM) inhibited HBV replication in one experiment and #40 (50 µM) inhibited replication in another experiment. Overt cellular toxicity was not observed for any of the compounds at 10 µM. Toxicity was often observed at higher concentrations; this led to the reduced yield of HBV DNA from cultures treated with 50 µM compounds #5, 6, and 8 in Fig. 10.

The effect of the compounds on replication of a genotype D isolate was tested to evaluate the generality of the results with the genotype A isolate. Treatment of capsid-derived nucleic acids from the DMSO control cells with exogenous RNAseH led to partial conversion of the double-stranded molecules to single-stranded forms. Therefore, RNA:DNA heteroduplexes accumulated in capsids even in the absence of RNAseH inhibitors. This indicates that the RNAseH activity during reverse transcription was incomplete for this isolate. Very few of the most slowly-migrating double-stranded nucleic acids accumulated in cells treated with 10 µM compound #12, and many of the duplex DNAs collapsed to single-stranded forms upon treatment with exogenous RNAseH. Therefore, the inefficient HBV RNAseH in this isolate created a high background, but we were able to detect suppression of the HBV RNAseH activity above background by compound #12. None of the other compounds tested against the genotype D isolate detectably inhibited HBV replication (Table 2).

Therefore, compound #12 inhibited replication of HBV genotypes A and D in cells at low µM concentrations by blocking RNAseH activity, with the anti-RNAseH effect being somewhat less pronounced than complete ablation of the activity by mutating the RNAseH active site.

## Discussion

Nucleos(t)ide analog therapy has turned chronic HBV infection into a disease that can be controlled indefinitely, with enormous benefits to patients [6], [7], [85]. However, the infection is very rarely cleared, so treatment is essentially life-long, very expensive, and may be associated with unpredictable long-term side effects. Despite these limitations, the ability of protracted nucleos(t)ide analog therapy to slowly suppress cccDNA and HBsAg and to cure a small minority of HBV patients [10]–[13], [21]–[23] indicates that the nucleos(t)ide analogs can push the virus to the brink of elimination. This implies that many more patients could be cured by employing a new drug against a novel HBV target in combination with the nucleos(t)ide analogs to further suppress HBV replication. Here, we report production of recombinant HBV RNAseH suitable for low throughput antiviral drug screening and demonstrate that chemical structure-activity relationships based on HIV RNAseH and integrase inhibitors can guide identification of compounds likely to inhibit the HBV enzyme.

Production of soluble recombinant HBV polymerase or domains of the polymerase is notoriously difficult, and our experience with the HBV RNAseH domain was no exception. Soluble HBV RNAseH accumulated to low levels in *E. coli* and was a minor component of the extracts even after nickel-affinity enrichment (Fig. 4). Much of the RNAseH was apparently cleaved near its N-terminus, and these cleavage products are unlikely to be active because their sizes imply that they lack D702. Although the concentration of the intact enzyme was very low, its specific activity was high enough to yield readily detectable signals in both radioactive and fluorescent RNAseH assays (Fig. 5). Potenza et al. [57] previously expressed recombinant HBV RNAseH that was very similar to HRHPL (genotype D), but their expression conditions led to accumulation of the enzyme in inclusion bodies, necessitating refolding following purification under denaturing conditions. The refolded enzyme possessed RNAse activity, but this activity was not demonstrated to be an RNAseH. Differences between the assays employed here and in Potenza's study prevent comparison of the specificity and specific activity of the enzyme prepared under native and denaturing conditions.

The optimal reaction conditions for the recombinant HBV RNAseH (Table 1) were typical for nucleic-acid modifying enzymes and were similar to conditions in which recombinant hepadnaviral reverse transcriptase is active [86]–[88]. Its activity was dependent upon a divalent cation, but it became active against single-stranded RNA in addition to RNA in a heteroduplex when Mn^++^ was substituted for Mg^++^ (data not shown). This is similar to the reduced fidelity of nucleic acid polymerases (including the duck HBV polymerase) in the presence of Mn^++^ [89]–[91]. The RNAseH had a relatively high NaCl optimum of 190 mM and it lost specificity for heteroduplex RNA at low ionic strength (data not shown). Importantly given that a primary goal of this study was to produce enzyme suitable for antiviral drug screening, recombinant HBV RNAseH was stable upon storage in liquid nitrogen, could be repeatedly frozen and thawed, and was fully active in up to 2% DMSO. Therefore, enzyme suitable for low-throughput anti-HBV RNAseH drug screening has been produced.

The HIV RNAseH is a very active target of ongoing antiviral drug discovery [38], [59]–[78], but to our knowledge none of the anti-HIV RNAseH compounds have entered clinical trials yet. This is primarily due to the relatively low therapeutic indexes of most known anti-HIV RNAseH compounds. Similar challenges were faced by the HIV integrase field in the early stages of development of anti-integrase drugs. Many inhibitors were discovered, but clinical development did not begin until strand transfer inhibitors, active site metal binders, etc. were discovered. The failure to advance to HIV RNAseH inhibitors to clinical trials may also be partially due to the large number, high potency, and diverse profile of existing anti-HIV drugs. In contrast, current anti-HBV therapies are primarily based on a single class of inhibitors, nucleos(t)ide analogs. Hence, inhibitors of a new HBV enzymatic function would address the current challenges of limited efficacy and cross-resistance among the nucleos(t)ide analogs, and this would allow meaningful combination therapies for HBV similar to HAART that dramatically changed the landscape of anti-HIV therapy.

The ability to template HBV RNAseH drug discovery on the HIV experience would greatly accelerate anti-HBV efforts. The HIV data could narrow the chemical space to be assessed during screening, compounds synthesized during anti-HIV RNAseH screening would be available for immediate screening against HBV, and the toxicity profile of some of these compounds is known. Templating anti-HBV RNAseH drug development on HIV efforts would be analogous to the development of the anti-HBV nucleos(t)ide analogs, which was greatly facilitated by the parallel development of anti-HIV nucleoside analogs [92].

Twenty-one candidate RNAseH inhibitors were selected due to their similarity to known inhibitors of the HIV RNAseH or integrase. Twelve of these compounds (57%) inhibited the HBV RNAseH at 10 µM to below the threshold defined by control reactions with irrelevant compounds (Fig. 7 and Table 2). Importantly, 10 of 11 compounds analogous to anti-HIV integrase compounds inhibited the HBV RNAseH, including both approved anti-HIV integrase drugs, raltegravir (compound #11) and elvitegravir (#10). This is consistent with the membership of both the RNAseH and integrase in the nucleotidyl transferase superfamily of enzymes. Therefore, there is enough similarity between the HBV RNAseH and the HIV RNAseH and integrase active sites to guide screening for anti-HBV RNAseH compounds.

Most anti-HIV RNAseH inhibitors bind to the enzyme and chelate the divalent cations in the active site [64], [65], [69], [70], [73], [76]. Similarly, anti-HIV integrase compounds that target the active site typically do so by binding to the enzyme or the enzyme plus DNA and chelating the active site divalent cations [93]. The compounds tested here were selected for the ability to bind to Mg^++^ ions oriented as they are in the HIV RNAseH or integrase active sites, and hence inhibition of the HBV enzyme is predicted to be through binding to the active site and interfering with the Mg^++^ ions. The mechanisms by which the HBV RNAseH inhibitors function have not been determined, but IC_50_ curves reveal at least two patterns. The profiles for compounds #12, 39, and 40 were consistent with the predicted competitive inhibition mechanism (Fig. 8C and data not shown). In these cases, inhibition appears to be specific. Other compounds, such as #6 and #8, had inhibition profiles with one or more broad plateaus that were inconsistent with simple competitive binding to the active site. In addition, the electrophoretic mobility of the RNA was retarded at high concentrations of compound #8 (Fig. 8C), implying that this compound may react with the RNA substrate.

The compounds employed here were selected by structure-activity relationships with the goal of testing whether these relationships could predict biochemical inhibition of the HBV RNAseH. The compounds were not selected to have other properties necessary for a drug, such as the ability to enter cells. Nevertheless, compound #12 inhibited HBV replication in cell culture at 10 µM without extensive cellular toxicity (Fig. 10). The reduction in mobility following treatment of capsid-derived nucleic acids with *E. coli* RNAseH demonstrates that RNA:DNA heteroduplexes accumulated in the viral capsid in the presence of compound #12, confirming that these compounds blocked HBV RNAseH activity in culture. Therefore, it is possible to pharmacologically inhibit the HBV RNAseH in cells, and identification of anti-HBV compounds that are active in cells can be achieved employing structure-activity relationships based on anti-HIV compounds. Furthermore, the ability of compounds identified by screening against recombinant genotype D and H enzymes to inhibit both genotype A and D isolates in culture demonstrates that it is possible to identify RNAseH inhibitors that are active against a range of HBV isolates.

The sensitivity profile of the HBV genotype D and H RNAseHs to the inhibitors was not the same (Table 2). This has two implications. First, the genotype H RNAseH may be a better candidate for primary drug screening than the genotype D enzyme because its inhibition profile more accurately predicted inhibition of HBV replication in culture. Second, the variable sensitivity of the genotype D and H enzymes to the compounds indicates that HBV's high genetic diversity is likely to be an important issue during development of anti-HBV RNAseH drugs.

The key HBV molecule that must be eradicated to cure patients is the viral cccDNA (Fig. 1) [25]. Ideally, clearing the cccDNA would be achieved by simultaneously suppressing its synthesis rate with the existing nucleos(t)ide inhibitors and increasing its degradation rate with a new drug. The problem with this approach is that we do not know how to safely destabilize the cccDNA, so the approach that has the most realistic chance of clearing HBV in the foreseeable future is to further suppress its synthesis rate. Importantly, pharmacological suppression of viral genomic synthesis may not need to completely eradicate the cccDNA by itself because the latter stages of viral clearance may be assisted by the immune system. HBV's proteins, including HBsAg [94]–[99], HBeAg [100], [101], and the polymerase [102]–[104], have immunosuppressive activities. Consequently, if viral genomic replication can be suppressed far enough to inhibit cccDNA synthesis rather than just virion secretion (Fig. 1) as is usually achieved with the nucleos(t)ide analogs, levels of the cccDNA would drop. This reduction in the transcriptional template would reduce production of HBV's proteins, presumably weakening HBV's immunosuppression and promoting immune-mediated viral clearance.

Three challenges remain prior to beginning full-scale antiviral drug screening against the HBV RNAseH. First, the majority of HBV's disease burden is caused by genotypes B and C, and we have been unsuccessful to date in generating consistently active recombinant RNAseH from these genotypes. This challenge is likely to be surmountable because only a few isolates of these genotypes have been tested for activity and because compound #12 identified by screening against genotypes D and H inhibited replication of HBV genotype A in culture, confirming that cross-genotype inhibition is possible. Second, the existing tissue culture and biochemical assays are sufficient for low throughput drug screening, but anti-HBV RNAseH drug development is anticipated to require screening many thousands of compounds even when the chemical search space is constrained by prior studies with HIV. Therefore, full-scale drug screening and subsequent mechanistic assessment of hit compounds will require improving the yield and purity of the biochemical RNAseH assay. This challenge should be met by further optimizing the induction and extraction conditions, expanding the bacterial induction cultures beyond the 100 ml scale used in this study, adding a second purification step such as ion-exchange chromatography, and expanding efforts to control proteolysis of the enzyme. We are optimistic this goal can be achieved because recent improvements to the induction and extraction conditions have increased the specific activity of the enzyme approximately four-fold, and initial scale-up experiments have not met with difficulty. Finally, the HBV RNAseH assay must be adapted to a format suitable for high throughput screening. This challenge should also be surmountable because fluorescent RNAseH assays have been widely employed to screen for anti-HIV RNAseH inhibitors and because the signal∶background ratio for the first-generation HBV RNAseH fluorescent assay in Fig. 5 should be improved by increasing the concentration of the RNAseH and/or by optimizing the substrate structure.

## Materials and Methods

### Plasmids and viral strains employed

pCMV-HBV-LE- (CMV-HBV) is an HBV over-length genomic expression vector containing 1.2 copies of the HBV(adw2) genome (Genbank X02763.1) downstream of the CMV promoter cloned into pBS (Promega). Surface protein expression from this vector is ablated by mutating the preS and S open reading frames. pCMV-HBV(genotype D) is an analogous HBV genomic expression construct and was a gift from Dr. Shuping Tong. For bacterial expression, codon-optimized cDNA sequences for HRHPL (genotypes A, B, C, D, and H) were cloned by gene synthesis (Genscript) between the NcoI and EcoRI sites into pTrcHis2B (Invitrogen) with a C-terminal hexahistidine tag. HRHPL contains HBV genotype D (Genbank V01460) polymerase residues 684–845. The human RNAseH1 gene (NP_002927.2) was cloned with an N-terminal hexahistidine-tag between the BamHI and XhoI sites of pRsetB (Invitrogen) by gene synthesis.

### RNAseH expression and enrichment

HRHPL and human RNAseH1 were expressed in *E. coli* BL21 codon+ cells (Invitrogen). Saturated overnight bacterial cultures were diluted 4-fold into 100 ml fresh medium and protein expression was induced with 0.5 mM IPTG at 30°C for six hours. The cells were lysed by sonication in lysis buffer [50 mM HEPES pH 8.0, 800 mM NaCl, 0.1% NP40, 27.5% glycerol, 2 mM DTT 20 mM imidazole, and protease inhibitor cocktail (Sigma)]. RNAseH proteins were enriched by nickel–agarose affinity chromatography, eluted with 350 mM imidazole, dialyzed into 50 mM HEPES pH 7.3, 300 mM NaCl, 20% glycerol, and 5 mM DTT, and stored in liquid nitrogen.

### *In vitro* RNAseH assays

For the oligonucleotide-directed RNAseH cleavage assay [46], 6 µl protein extract (typical protein concentration 2.8 mg/ml) was mixed with 0.5 µg internally ^32^P-labeled DRF+ RNA (nucleotides 2401–2605 of the duck HBV genome plus 60 nucleotides of flanking sequences from pBluescript) and 3 µg oligonucleotide D2507− or its corresponding negative control D2526+ on ice in 20 µl under the conditions in Table 1. Some reactions in Fig. 5 employed oligonucleotide D2543M-Sal or its D2453+ negative control as indicated. The reactions were incubated at 42°C for 90 min. and terminated by addition of Laemmli protein loading buffer and boiling. The samples were resolved by 12% SDS-PAGE, the gels were stained with Coomassie blue to monitor protein loading, and labeled RNA was detected by autoradiography. Candidate inhibitors were dissolved in DMSO and added at the indicated concentrations during assembly of the reactions. Control reactions lacking the compounds contained DMSO as a vehicle control. The RNAseH autoradiograms were scanned and quantified with ImageJ. The oligonucleotides were: D2526+ (CCACATAGGCTATGTGGAAC), D2507− (GTTCCACATAGCCTATGTGG), D2453+ (CCGCCTGATTGGACGGCTTTTCC), and D2543M-Sal (GCAACTGTGTCGACAGCAGCTCCGAAGGAGA).

For the fluorescent RNAseH assay, the DRF+ RNA and DNA oligonucleotides were omitted from the RNAseH reactions and replaced with 20 µM of the quenched fluorescent chimeric RNA:DNA oligonucleotide RHF1; the reaction conditions were identical to the oligonucleotide-directed reactions except that the NaCl concentration was reduced to 130 mM. The reactions were incubated in the dark at 42°C for 90 min. prior to termination by addition EDTA to 10 mM and detection of fluorescence at 520 nM on Synergy 4 plate reader (Biotec, Inc.). The sequence of the RHF1 substrate (IDT, Inc.) was: 5′-56-FAM/rCrCrArCrArUrArGrGrCrUrArUrGrUrGrGrArArCTTTTGTTCCACATAGCCTATGTGG/3IBkFQ/-3′. The RNA:DNA heteroduplex in the RHF1 substrate was the same as the heteroduplex formed by oligo D2507− annealed to DRF+.

### Cell-based HBV replication inhibition assays

Huh7 cells were maintained in Dulbecco's modified Eagle's medium with 10% fetal bovine serum at 37°C in 5% CO_2_. Cells were seeded into 60 mm dishes and transfected at 70% confluency with 2.6 µg of plasmids using TransIT-LT1 (Mirus, Inc.). Test compounds were added the morning following transfection at 10 or 50 µM, and fresh medium containing the compounds was provided every 1–2 days. Four or five days post-transfection HBV cores were isolated by lysis of the cells in 10 mM Tris pH 7.5, 1 mM EDTA, 0.25% NP40, 50 mM NaCl, and 8% sucrose followed by sedimentation through a 30% sucrose cushion as described [105]. Viral DNAs were isolated from cytoplasmic core particle preparations by proteinase K digestion followed by phenol/chloroform extraction as described [46]. Duplicate aliquots of the nucleic acids were treated with 2 U *E. coli* DNAse-free RNAseH (Invitrogen) at 37°C for 30 min. or were mock treated. The nucleic acid samples were resolved by electrophoresis on 1.2% agarose gels and detected by Southern blotting with ^32^P-labeled HBV DNA as a probe.

## Supporting Information

Figure S1 **Chemical structures of the compounds tested.** Compounds are named by the company/product number or their formal names, as appropriate. The approved anti-HIV integrase drugs Elvitegravir (#10) and Raltegravir (#11) are listed by their common names, pharmaceutical developer's codes, and company/product numbers. Compound #40 is listed by its NIH AIDS Research and Reference Reagent Program number.
(PDF)

Table S1 **Residual activity in RNAseH reactions conducted in the presence of 10 µM of the test compounds.** Values are normalized to vehicle control reactions containing 1% DMSO and the error ranges are ± the standard deviation from 3 to 7 replicate experiments.
(PDF)
