# Supplementary material for: The Hepatitis B Virus Ribonuclease H Is Sensitive to Inhibitors of the Human Immunodeficiency Virus Ribonuclease H and Integrase Enzymes
Source: PLoS Pathog. 2013 Jan 22;9(1):e1003125. doi: 10.1371/journal.ppat.1003125 (PMC3551811; doi:10.1371/journal.ppat.1003125)
Supplement: Figure S1 — Chemical structures of the compounds tested. Compounds are named by the company/product number or their formal names, as appropriate. The approved anti-HIV integrase drugs Elvitegravir (#10) and Raltegravir (#11) are listed by their common names, pharmaceutical developer's codes, and company/product numbers. Compound #40 is listed by its NIH AIDS Research and Reference Reagent Program number. (PDF) [file ppat.1003125.s001.pdf]

Supplemental Figure 1.

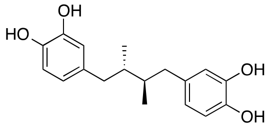

2
Sigma 74540

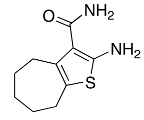

3
Sigma n8164

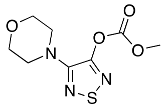

4
Tim Tec ST029023

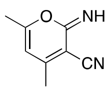

5
Enamine T0506-3483

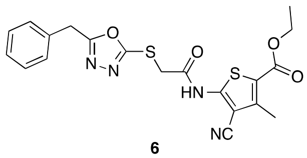

6
Chembridge 7929959

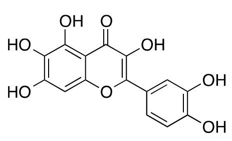

7
Indofine 021030

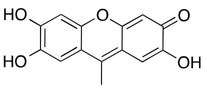

8
Sigma S439274

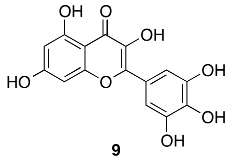

9
Sigma 70050

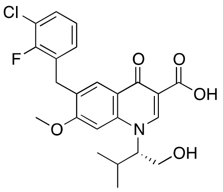

10
Selleck Chemicals S2001
GS9137 (elvitegravir)

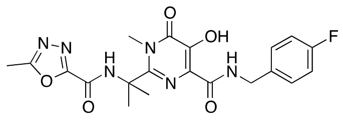

11
Selleck Chemicals S2005
MK0518 (raltegravir)

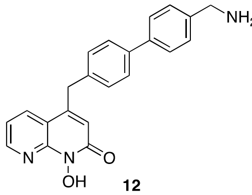

12
NAPHRHI

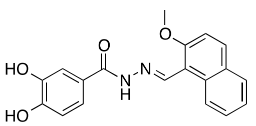

13
DHBNH

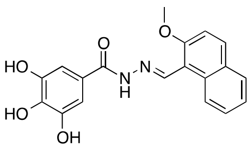

14
THBNH

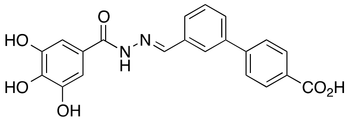

15
BHMP07

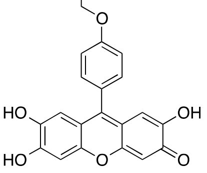

30
Chembridge 7248520

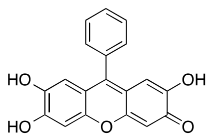

31
ChemBridge 5104346

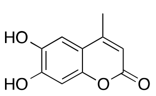

34
Indofine D-009

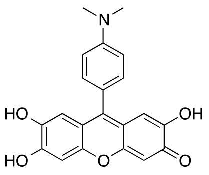

35
TCI America D1118

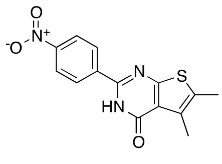

38
Vistas M Lab STK317995

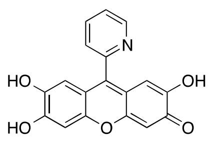

39
Asinex BAS0223612

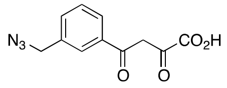

40
118-D-24
