# Supplementary material for: The Hepatitis B Virus Ribonuclease H Is Sensitive to Inhibitors of the Human Immunodeficiency Virus Ribonuclease H and Integrase Enzymes
Source: PLoS Pathog. 2013 Jan 22;9(1):e1003125. doi: 10.1371/journal.ppat.1003125 (PMC3551811; doi:10.1371/journal.ppat.1003125)
Supplement: Table S1 — Residual activity in RNAseH reactions conducted in the presence of 10 µM of the test compounds. Values are normalized to vehicle control reactions containing 1% DMSO and the error ranges are ± the standard deviation from 3 to 7 replicate experiments. (PDF) [file ppat.1003125.s002.pdf]

Supplemental Table 1.

	Residual activity at 10 uM[1]	
**Compound**	**Genotype D**	**Genotype H**
2	54±18	67±20
3	43±14	68±14
4	35±6	68±18
5	42±15	76±14
6	40±16	81±24
7	45±19	68±24
8	33±17	74±21
9	33±10	66±23
10	30±8	66±21
11	29±18	63±21
12	28±12	17±6
13	52±12	42±26
14	46±20	27±13
15	47±4	37±23
30	23±6	37±8
31	26±15	50±10
34	30±15	33±12
35	27±14	38±20
38	24±1	38±25
39	21±2	26±0
40	21±16	22±2

[1] Percent DMSO control ± standard deviation
